# Supplementary figures and images for: Music-Based Intervention Ameliorates Mecp2-Loss-Mediated Sociability Repression in Mice through the Prefrontal Cortex FNDC5/BDNF Pathway
Source: Int J Mol Sci. 2021 Jul 2;22(13):7174. doi: 10.3390/ijms22137174 (PMC8269182; doi:10.3390/ijms22137174)

Supplementary Materials

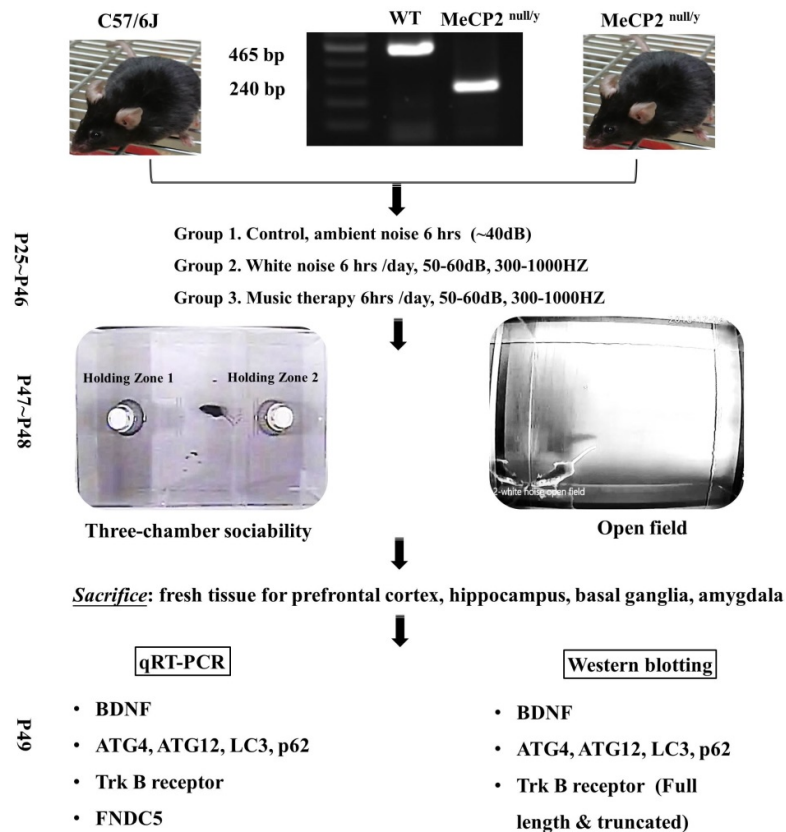

Supplementary Figure S1. Illustrative flow chart of the study design

Supplement: Supplementary file 1 [file ijms-22-07174-s001.zip › ijms-1269946-supplementary.pdf]
